# Population- and Gender-Based Investigation for Prevalence of *Helicobacter pylori* in Dhamar, Yemen

**DOI:** 10.1155/2023/3800810

**Published:** 2023-01-25

**Authors:** Dhary A. Almashhadany, Sara M. Mayas, Hero I. Mohammed, Abdulwahed A. Hassan, Izhar U. H. Khan

**Affiliations:** ^1^Department of Medical Laboratory Science, College of Science, Knowledge University, Erbil 44001, Iraq; ^2^Department of Biology, Collage of Applied Science, Thamar University, Dhamar, Yemen; ^3^Department of Veterinary Public Health (DVPH), College of Veterinary Medicine, University Mosul, Mosul, Iraq; ^4^Agriculture and Agri-Food Canada, Ottawa Research and Development Centre, 960 Carling Ave. Ottawa, ON K1A 0C6, Canada

## Abstract

Among 35 species of genus *Helicobacter*, *H. pylori* is the most common causative agent of human gastritis, peptic ulcer, and gastric cancer. The infection can spread through direct human-to-human contact, fecal–oral route, and contaminated water. The study was designed to investigate the rate of prevalence of *H. pylori* in the population of Dhamar, Yemen. In this one-year study, 460 including 250 male and 210 female stool specimens were collected between January to December 2020 in Dhamar Governorate, Yemen. Of the total 460, 215 rural (male: *n* = 120 and female: *n* = 95) and 245 urban (male: *n* = 130 and female: *n* = 115) specimens were investigated for identification of *H. pylori* by serological test using *Helicobacter pylori* stool antigen (HpSA) test. In addition, for comparing an improved recovery of *H. pylori*, conventional culture-based isolation was also carried out using three selective media. Modified Campy-blood Agar (MCA), Belo Horizonte Agar (BHA), and Egg yolk Emulsion (EYE) medium supplemented with antimicrobial agents including vancomycin (10 mg/L), cefsulodin (5 mg/L), trimethoprim (5 mg/L), and amphotericin B (5 mg/L) and isolates were phenotypically characterized. The HpSA test results revealed that of the total 460 specimens, 89 (19.3%) were positive for *H. pylori* with relatively low in male (*n* = 43; 17.2%) as compared to the female (*n* = 46; 21.9%) specimens. After 3–10 days of incubation, *H. pylori* was recovered at a variable rate on each selective (MCA: 16.5%; BHA: 15.0%; EYE: 13.0%) media. However, culture-based assay results showed less recovery (*n* = 81; 17.6%) with no significant difference among all selective media tested and between genders (male: *n* = 39; 15.6%; female: *n* = 42; 20.0%). The infection rate was comparatively higher in rural (*n* = 45; 20.9%) as compared to urban (*n* = 36; 14.7%) population. Overall, the study data showed the prevalence of infection in both genders of all age groups. The present study showed a relatively high rate of infection of *H. pylori* in the Dhamar population. The serological identification and culture-based methods are important for rapid detection, aid in treatment, and developing policies for the control and eradication of *H. pylori* infection and to prevent the disease in different age groups in Yemen.

## 1. Introduction

The species of genus *Helicobacter* are microaerophilic, motile, and Gram-negative curved rods bacterium that belongs to the family *Helicobacteraceae*. Currently, the genus consists of over 30 species that have been classified into two major lineages: gastric and nongastric or enterohepatic [1]. The gastric lineage represents only one-third of all *Helicobacter* species that colonize in the stomach of humans and a wide range of animal species e.g., cattle, sheep, cats, dogs, monkeys, cheetahs, rhesus, ferrets, dolphins, and whales. Among eight *Helicobacter* species of gastric group, *H. pylori* (∼2–3.5 *μ*m × 0.5 1.0 *μ*m) is the representative species of medical importance and considered as a causative agent of active chronic gastritis, peptic and duodenal ulcer, gastric cancer, and mucosa-associated lymphoid malignancies in humans [2, 3]. In the last decades, an increasing number of studies have performed an analysis of the gastric mucosa to identify the role of *H. pylori* in peptic ulcer, carcinogenesis, and in some forms of gastric lymphoma [4]. It has been observed that *H. pylori* colonizes under the coating layer of the gastric pits close to gastric epithelial cells and in the gastric mucin covering [1, 4]. However, the enterohepatic or nongastric *Helicobacter* species have been more frequently identified in the human intestinal tract and liver, other mammals (e.g., rodents, rats, mice, and hamsters), and birds. In many cases, they have been linked with inflammation or malignant transformation in immunocompetent hosts and more severe clinical diseases in immunocompromised humans and animals [1, 5].

The prevalence of *H. pylori* infection has been considered as one of the most common infections in both developing and developed countries with an estimated 4.4 billion cases reported in 2015 [3]. Clinically, over 80% of cases are asymptomatic [6]. The prevalence of *H. pylori* infection ranged between 7.3 and 92.0%, depending on different factors such as geographic location, age, and socio-economic status [7, 8]. The prevalence in Asia, South America, and South and East Europe is ˃50%, whereas only one-third of population has been reportedly infected in North American and North European countries [9]. Previous epidemiological studies have reported more *H. pylori* infection in developing countries (up to 90%) in some regions such as Bhutan and Myanmar [10, 11]. The socio-economic and hygiene status have been considered as main risk factors for infection where high prevalence rates have been reported in crowded areas, especially in rural areas with low socio-economic and unhygienic conditions [10, 12].

Several diagnostic methods have been developed and applied for the identification and characterization of *H. pylori*. Invasive diagnostic tests including endoscopic imaging, histology (microscopy), rapid urease test, and culture methods are mostly used to obtain biopsy materials to aid in the treatment of infection [13, 14]. However, the most common noninvasive diagnostic assays, including stool antigen tests (SATs), serology-based tests, urea breath tests, and molecular assays have been widely applied [15].

Previous epidemiological studies of *H. pylori* in Yemen population have not been well-reported [7, 16–18]. Therefore, the present population- and gender-based study was designed to detect and identify the rate of prevalence of *H. pylori* infection in urban and rural male and female patients in the city of Dhamar, Yemen. Moreover, we also assessed and compared the rate of recovery of *H. pylori* on three selective media, especially when cell concentration is low in the specimen.

## 2. Materials and Methods

### 2.1. Specimen Collection

A total of 460 human stool specimens were collected between January to December 2020 from General Dhamar Hospital and different medical diagnostic laboratories located in Dhamar Governorate, Yemen. The stool specimens were collected from 250 males (rural: 120; urban: 130) and 210 females (rural: 95; urban: 115). The age of the participants ranged from <1 to 80 years.

### 2.2. Detection of *H. Pylori*

#### 2.2.1. *H. Pylori* Antigen Screening

The principle of the *H. pylori* stool antigen (HpSA) test is a noninvasive diagnostic module based on immunochromatography (ICA) assay for *H. pylori* infection where One-Step*H. pylori* antigen test card (LumiQuick, Santa Clara, USA) was used. Briefly, approx. 200 mg of stool sample was transferred into a vial containing 2 mL phosphate buffer saline (PBS), the solution was shaken for 30 seconds and then 120–150 *μ*L of homogenous suspension was loaded in the well of One-Step*H. pylori* antigen test card strip (LumiQuick, USA). The results were recorded after 10–15 min based on a red line developed at the C&T region, whereas a red line developed at the C band was recorded as negative as described by the manufacturer.

#### 2.2.2. Culture-Based Isolation

Fresh stool specimen was incubated at 37°C in a humid atmosphere enriched with CO_2_ (∼12%) for 2 hr. Then, ∼0.5 g of stool was transferred into a sterile tube containing 15 mL of *Brucella* broth (Merck, Darmstadt, Germany), supplemented with 20% glycerol and 0.5 g cholestyramine (a basic anion exchange resin that binds bile acids). The mixture was homogenized by vortexing and 100 *μ*L of homogenized suspension was streaked on three selective agar media: (1) Modified Campy-blood Agar (MCA) medium composed of Brucella Agar base (Oxoid, Wesel, Germany) with 10% sheep blood; (2) Belo Horizonte Agar (BHA) medium contained Brain–Heart Infusion base (0.4%), 3,4,5-triphenyltetrazolium chloride (0.4%), and sheep blood (10%); and (3) Egg Yolk Emulsion (EYE) medium (Oxoid) contained Columbia Agar, supplemented with 10% egg yolk emulsion, 1% Vitox, and 2,3,5-triphenyltetrazolium chloride (40 mg/L) (Oxoid). All three media were supplemented with vancomycin (10 mg/L), cefsulodin (5 mg/L), trimethoprim (5 mg/L), and amphotericin B (5 mg/L) (Oxoid) [19]. The plates were incubated at 37°C for 3–10 days under microaerophilic condition where 100% humidity was provided in a candle jar as previously described [20].

#### 2.2.3. Cultivation and Species Identification

The putative *H. pylori* culture isolates recovered from each selective growth media were identified based on colony morphology, Gram staining reaction, and microscopic characteristics, including S-shaped or curved thin rods. Similarly, a panel of biochemical tests including catalase, oxidase, urease, indole, and growth in 1% glycine and 3.5% NaCl were performed. Single colonies were subcultured onto blood agar, incubated at 37°C for 48–72 h and stored at −20°C in cysteine storage medium containing 20% glycerol for further analysis.

### 2.3. Antimicrobial Susceptibility Testing

Antimicrobial susceptibility test was performed using disc diffusion (Kirby–Bauer) method [21]. Two types of antibiotics, nalidixic acid (30 *µ*g) and cephalothin (30 *µ*g) were used as described by the manufacturer (HiMedia, Düsseldorf, Germany). The *H. pylori* cell suspension was prepared in 2 mL sterile normal saline at an inoculum concentration equivalent to McFarland 3, where 10 *µ*L was spread plated on Columbia agar media. The plates were incubated under microaerophilic conditions at 37°C for 48–72 h using an anaerobic candle jar. The zone of inhibition was measured to classify them as sensitive or resistant according to the CLSI (2012) and CASFM (2017) guidelines and interpretative criteria.

### 2.4. Questionnaire

All patients investigated in this study were enrolled based on their clinical symptoms and history of treatment including antibiotics or other medications. A computer-assisted questionnaire including demographic and socio-economic information (age, gender, birthplace, education, and household population), clinical symptoms (chronic gastritis, gastric ulcer and/or duodenal ulcer, and stomach pain), any antibiotic and/or other treatment, and other factors (smoking and alcohol) was completed.

### 2.5. Statistical Analyses

Data was analysed using SPSS statistical software, version 25. Confidence intervals were calculated by the normal approximation method and *α*-level was set to 0.05. The Chi-square test was used to evaluate differences between groups and *p* < 0.05 was considered as significant.

## 3. Results

### 3.1. Serological Screening of *H. Pylori*

The HpSA screening assay of 460 stool specimens showed an overall relatively low frequency (*n* = 89; 19.3%) of *H. pylori* with comparatively high frequency in rural (*n* = 49; 22.8%) as compared to urban (*n* = 40; 16.3%) specimens where an insignificant (*p* > 0.05) difference was observed. However, a low frequency in males (*n* = 43; 17.2%) than in females (*n* = 46; 21.9%) with an insignificant (*p* > 0.05) difference was recorded in these specimens. On the other hand, when a comparative analysis between rural and urban male/female was preformed, *H. pylori* was detected more frequently in rural male (*n* = 24; 56%) and female (*n* = 25; 54%) than in urban male (*n* = 19; 44%) and female (*n* = 21; 46%) specimens. However, there was no significant (*p* > 0.05) difference found (Table 1).

### 3.2. Comparison and Assessment of Culture-Based Assays for Recovery and Detection

Similar to HpSA test results, *H. pylori* was recovered, between 3–10 days, from all three selective growth media with relatively low frequency (*n* = 81; 17.6%) in male (*n* = 39; 15.6%; rural: 22 and urban: 17) and female (*n* = 42; 20%; rural: 23 and urban: 19) specimens. Similar to serological test results, comparatively a high frequency of *H. pylori* positive cultures was recovered from rural males (*n* = 22; 49%) and females (*n* = 23; 51%) than in urban males (*n* = 17; 47%) and females (*n* = 19; 53%) with no significant (*p* > 0.05) difference (Table 1). Of three selective growth media, *H. pylori* cultures were isolated at a variable frequency on MCA (16.5%), BHA (15.0%), and EYE (13.0%) media with no significant (*p* > 0.05) difference. The cells were slow growing with small and translucent colony morphology. Microscopically, the Gram-negative colonies appeared as spiral-shaped rods. A slow cell growth was observed on blood agar base at 37°C under anaerobic condition. *H. pylori* cells grown on MCA media were small, round, and appeared translucent due to the blood agar background. Whereas golden colony morphology was observed on BHA due to 2,3,5-triphenyltetrazolium chloride as compared to EYE media where small red colony morphology was observed due to egg yolk agar base containing oxidation-reduction indicator (tetrazolium red) that changed yellow to red. *H. pylori* colonies on EYE media were distinctively visible, and confluent growth was very obvious against the yellow background. Based on the colony morphology observed on three selective agar media, BHA had more visible and distinctive (translucent golden) colonies followed by EYE and MCA media.

All culture isolates recovered from three selective culture media showed positive biochemical reactions to catalase, oxidase, urease, and TSI with Pb acetate paper tests and negative to indole. Moreover, isolates were positive for H_2_S production and hippurate hydrolysis test. The culture showed clear growth in 1% glycine and Brucella broth with 3.5% sodium chloride. All culture isolates (*n* = 81) showed resistance to nalidixic acid (30 *µ*g) and sensitivity to cephalothin (30 *µ*g).

### 3.3. Population-Based Assessment of *H. Pylori* Infection

A total of 460, including 250 (54.3%) male (rural: 120; urban: 130) and 210 (45.7%) female (rural: 95; urban: 115), participants involved and completed the questionnaires for this study. All patients were further investigated based on their clinical symptoms such as stomach pain, heartburn and various degree of nausea, vomiting, loss of appetite, and sometimes headache. The patients were not treated with antibiotics or other medication at least two months prior to visiting university's internal diseases clinic.

Overall, prevalence of *H. pylori* in Dhamar population was ranged from 15.74% to 22.96% (95% CI). According to antigen and cultural-based results, Table 1 shows the gender group-based rate of prevalence of *H. pylori* for all specimens investigated in the study. Although the frequency of *H. pylori* in females was relatively high, no gender-associated significant (*p* > 0.05) correlation with *H. pylori* infection was observed in both serological and culture-based assay results.

**Figure 1:**
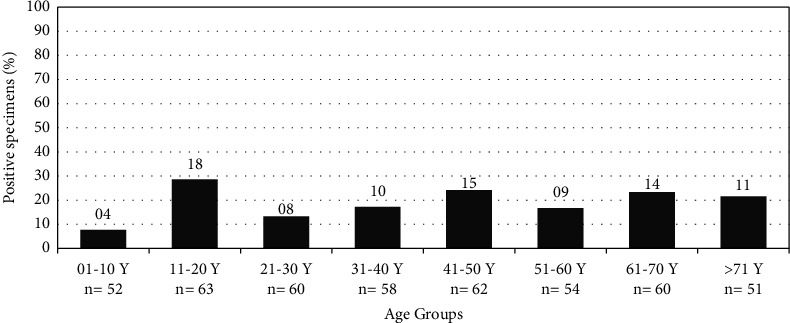
Rate of prevalence of *H. pylori* based on age groups with 10 years interval. The number of positive specimens is shown above each bar.

Moreover, the rate of prevalence among various participants' age groups ranged from <1 to 80 years was assessed. The highest prevalence rate (28.6%) was observed among adolescents. In contrast, *H. pylori* infection among children <11 year was significantly (*p* < 0.05) lower than other age groups (Table 2). The detailed distribution of positive cases is graphically depicted in Figure 1 based on a 10-year-age interval. Regarding the residence area, *H. pylori* prevalence among participants from rural was higher than urban areas. However, this difference was not statistically significant (*χ*^2^ = 1.05, *p* > 0.05).

**Figure 2:**
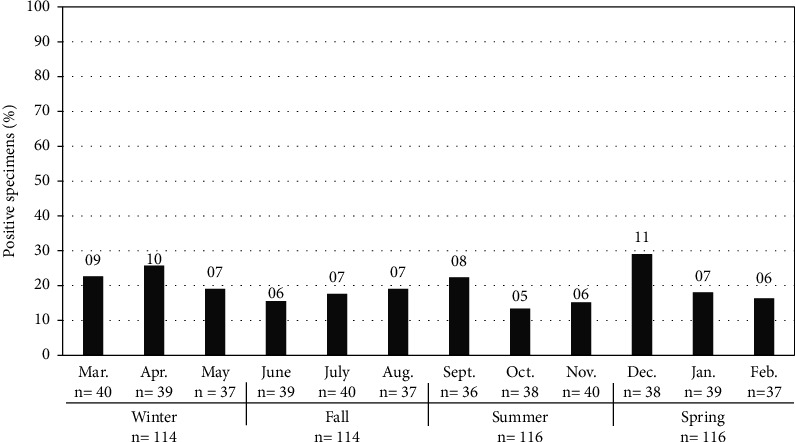
Seasonal and monthly rate of prevalence of *H. pylori* where number of positive specimens is shown above each bar.

The data analysis was further performed to assess the seasonal occurrence of *H. pylori* infection where the distribution of *H. pylori* cases was monitored for a full year (Figure 2). The highest prevalence in December (28.9%) while the lowest rate in October (13.2%) with no significant (*p* > 0.05) seasonal difference was recorded. Based on the questionnaire data results, the source of drinking water and household population did not show significant (*p* > 0.05) difference. Moreover, 78% of participants showed asymptomatic positive cases of *H. pylori* infection.

## 4. Discussion

Since *H. pylori* infection has been related to gastritis, peptic ulcers, and gastric cancer [22], therefore, epidemiological surveillance of *H. pylori* helps in establishing public health counter measures that could reduce and control the transmission and acquisition of infection [23]. A variable (ranged between 30 and 80%) rate of prevalence of *H. pylori* infection between developed and developing countries has been reported [24]. As compared to 6 to 12% in developed countries (Japan, Germany, Netherlands, and USA), high infection rate (26 to 66%) has been reported in developing (Chile, Venezuela, and Nigeria) countries [25]. The rate of frequency (19.3%), in our study, is similar to the previous surveillance studies (ranged 12 to 18%) conducted in countries (Lebanon, Egypt, Saudi Arabia, and Iran) located in the same geographical area [26–29]. In contrast, high prevalence rates (ranged from 40 to 82%) were also reported in the same area [30–33]. This serological variable data could be due to numerous contributing factors such as socio-economic status, household population, hygienic lifestyle, living in crowded families, and family history of GI cancer that might impact on the prevalence rate of *H. pylori*.

Moreover, the detection methods are also one of the major contributing factors [11, 34, 35]. Although, no single test can be considered as the gold standard [36]. Generally, there are two (enzyme immunoassay (EIA) and immunochromatography assay (ICA)) types of SATs for the diagnosis of *H. pylori* infection using either mono- or polyclonal antibodies. Although both tests are highly sensitive and specific, the EIA-based tests appear to be more accurate than the ICA-based tests, but the ICA-based tests do not require special equipment, easy to use and useful for rapid diagnosis of *H. pylori* infection [37]. *H. pylori* stool antigen (HpSA) test is a rapid assay applied for rapid screening and diagnostics since it is accurate, sensitive, economical, and simple. However, the results may be influenced by some factors such as the amount of antigen in stool, the nature of biochemical reaction in the kit, and the heterogeneity of antigens [34]. Urea breath test (UBT) and stool antigen test (SAT) have been widely applied for the detection of *H. pylori* infection. Since the sensitivity and specificity of SAT ranged between >80% and >90% [38], Canadian Agency for Drugs and Technologies in Health extensively reviewed SAT test for *H. pylori* detection and approved due to its diagnostic accuracy, clinical, and cost effectiveness [37]. In recent meta-analysis study, the pooled sensitivity and specificity SATs test results between 92 and 94% [39]. Similarly, Schulz et al. [40] evaluated nine different strategies for screening of *H. pylori*-infected asymptomatic immigrants and refugees to Australia. Stool antigen screening and repeat testing were found the most cost-effective approach for reducing the future burden of peptic ulcer (PU) and gastric cancer in the population. Previous studies have evaluated indirect methods, including antibody-based tests (serology and urine test), UBT, and SAT that have been used for population-based screening for detecting *H. pylori* infection.

Similar to previous research studies, our study results have showed no significant correlation associated with gender-based (male and female) distribution of *H. pylori* infection [14, 41]. The data indicates that gender has a negligible role in the *H. pylori* infection. Also, rate of prevalence in rural and urban area results did not show significant difference. In contrary, based on age group data analysis, a significant difference in the prevalence of *H. pylori* infection where low (7.7%) frequency in age group between 1 and 9 years as compared to age group between 10 and 19 years with a high (28.6%) frequency was recorded. Our results are incongruence with previous studies where high infection rate was reported after childhood as compared to other studies where *H. pylori* infection was reported during childhood and adolescence [42–46]. On the other hand, other studies reported high prevalence in children regardless the differences in the associated risk factors [27, 47]. Interestingly, increased rates of infection, as well as spontaneous recovery was observed primarily in children under 5 years [48]. In a recent study, Park et al. [25] reported that *H. pylori* infection in children and adolescents was not significantly associated with age and gender.

We also assessed seasonal association with *H. pylori* prevalence. The statistical analysis showed no significant (*p* > 0.05) difference between seasons. These findings indicate the independency of *H. pylori* prevalence regardless the season. However, previous study showed an increase of infection frequency from 70% in the dry months to 96% in the rainy months among youngsters only, while incidence pattern in adults showed no statistical association with season [49]. Similarly, *H. pylori* infection frequency was increased in February (40%) and declined afterwards [7]. The participants of this study used tap water pipe as drinking water source, therefore, showed no significant differences between the populations. On the contrary, other previous studies found a significant correlation between the source of drinking water and *H. pylori* infection [14, 50].

A comparative analysis among three selective media was performed to assessthe enhanced and improved recovery of *H. pylori* from both urban and rural male and female patients. Generally, detection of *H. pylori* from stool samples showed low (18%) recovery rate [51]. However, Dore et al. [52] successfully recovered *H. pylori* (26.3%) from stool of known *H. pylori* infected patients after treating stool specimens with cholestyramine before plating on culture medium. Other studies recovered *H. pylori* between 15 and 39.1% of stool specimens [53, 54]. According to Alsulaimany et al. [38], the cultural method showed sensitivity ranged between 63 and 90% and specificity between 93 and 100%. These variations are mostly linked to factors such as specimens and/or culture media and incubation conditions as well as the stage of infection [35, 51]. Indeed, the density of bacteria in the stomach is an important determinant of successful recovery of *H. pylori* from stool. Additionally, the use of cholestyramine in the initial treatment of stool samples improves the yield of *H. pylori* in cultural methods, which may be related to the successful isolation of the metabolically active form of *H. pylori * [35].

In addition, we also investigated antimicrobial resistance of these culture isolates, where all *H. pylori* strains showed resistance to nalidixic acid and sensitivity to cephalothin with no statistically significant difference between male and female rural and urban specimens. Similar results were reported by Meng et al. [55], where variable drug resistance to metronidazole, clarithromycin, amoxicillin, levofloxacin, and furazolidone with no statistically significant difference between different sex, age, and disease category was reported. Thus, the antibiotic resistance of *H. pylori* could be considered a risk factor for the development of gastric cancer [56]. However, it has been recently reported that rabeprazole–bismuth–tetracycline–tinidazole regimen is a highly effective treatment for *H. pylori* infection where men have higher eradication results than women [57]. Further research is warranted to investigate the mechanism of action of these compounds that variably impact on the treatment of *H. pylori* infection in each gender.

## 5. Conclusion

The study reports on population- and gender-based rate of *H. pylori* prevalence and infection in Dhamar governorate, Yemen. Overall, *H. pylori* infection was detected at a relatively low level in the area and in both genders. It is not conclusive whether gender is a risk factor for the increased prevalence seen among females. Similarly, residents of rural areas may become more infected due to low socio-economic status and level of education, as well as suboptimal sanitary conditions and increased susceptibility as a result of a genetic tendency. Temporal impact on *H. pylori* infection was not observed as no statistical differences across seasons were found. Personal hygiene and proper food cooking and processing are recommended to reduce the spread of *H. pylori* infections. Further epidemiological molecular characterization is warranted to understand the transmission pathway in order to develop and improve strategies to control infection in the community.

## Figures and Tables

**Table 1:** Number (percent) of *H. pylori* positive specimens using strip One-Step* H. pylori* antigen test and selective cultural media.

Detection methods	Sampling source	Total no. of specimens	No. of positive specimens (%)	Statistical values			
				X̄	SD	95% CI	
						Upper limit	Lower limit
Serological test	M	250	43 (17.2)	146.5	146.4	186.3	106.7
		R: 120	R: 24 (20)	72	67.9	90.5	53.5
		U: 130	U: 19 (14.6)	74.5	78.5	95.8	53.2
	F	210	46 (21.9)	128	116.0	159.5	96.5
		R: 95	R: 25 (26.3)	60	49.5	73.5	46.5
		U: 115	U: 21 (18.3)	68	66.5	86.1	49.9
	T	460	89 (19.3)	274.5	262.3	345.8	203.2
	R	215	49 (22.8)	132	117.4	163.9	100.1
		M: 120	M: 24 (20)	72	67.9	90.5	53.5
		F: 95	F: 25 (26.3)	60	49.5	73.5	46.5
	U	245	40 (16.3)	142.5	145	181.9	103.1
		M: 130	M: 19 (14.6)	74.5	78.5	95.8	53.2
		F: 115	F: 21 (18.3)	68	66.5	86.1	49.9
	T	460	89 (19.3)	274.5	262.3	345.8	203.2

Cultural and biochemical tests	M	250	39 (15.6)	144.5	149.2	185.1	103
		R: 120	R: 22 (18.3)	71	69.3	89.8	52.2
		U: 130	U: 17 (13.1)	73.5	79.9	95.2	51.8
	F	210	42 (20)	126	118.8	158.3	93.7
		R: 95	R: 23 (24.2)	59	509	72.8	45.2
		U: 115	U: 19 (16.5)	67	67.9	85.5	48.5
	T	460	81 (17.6)	270.5	268	242.3	197.7
	R	215	45 (20.9)	130	120.2	162.7	97.3
		M: 120	M: 22 (18.3)	71	69.3	89.8	52.2
		F: 95	F: 23 (24.2)	59	50.9	72.8	45.2
	U	245	36 (14.7)	140.5	147.8	180.7	100.3
		M: 130	M: 17 (13.1)	73.5	79.9	95.2	51.8
		F: 115	F: 19 (16.5)	67	67.9	85.5	48.5
	T	460	81 (17.6)	270.5	268	243.3	197.7

R: rural; U: urban; M: male; F: female; T: total samples; (statistic value = *X̄*: sample means (average); SD: standard deviation; CI: confidence interval).

**Table 2:** Serological data based on socio-demographic and socio-economic parameters used for prevalence of *H. pylori* in Dhamar population.

Parameters		No. of specimens	No. of positive specimens (%)	Statistical values			
				X̄	SD	95% CI	
						Upper limit	Lower limit
Age group (years)	1. (01–10)	52	04 (7.7)	28	33.9	37.2	18.8
			M: 2	27	35.4	36.6	17.4
			F: 2	27	35.4	36.6	17.4
	2. (11–20)	63	18 (28.6)	40.5	31.8	49.1	31.9
			M: 8	30	38.9	39.6	20.4
			F: 10	31	29.7	38.3	23.7
	3. (21–30)	60	8 (13.3)	34	36.7	43.2	24.0
			M: 3	31.5	40.3	41.7	21.3
			F: 5	32.5	38.9	42.3	22.7
	4. (31–40)	58	10 (17.2)	34	33.9	43.2	24.7
			M: 4	31	38.2	40.8	21.2
			F: 6	32	36.8	41.5	22.5
	5. (41–50)	62	15 (24.2)	38.5	33.2	47.5	29.5
			M: 7	34.5	38.9	44.2	24.8
			F: 8	35	38.2	44.5	25.5
	6. (51–60)	54	9 (16.7)	31.5	31.8	40.1	22.9
			M: 6	30	33.9	39.1	20.9
			F: 3	28.5	36.1	38.1	18.9
	7. (61–70)	60	14 (23.3)	37	32.5	45.8	28.2
			M: 7	33,5	37.5	43	24.0
			F: 7	33.5	37.5	43	23.9
	8. (>71)	51	11 (21.6)	31	28.3	38.7	23.3
			M: 5	28	32.5	36.9	19.1
			F: 6	28.5	31.8	37.2	19.8
	T	460	89 (19.3)	274.5	262.3	345.8	203.2
			M: 43	251.5	294.9	278.4	224.6
			F: 46	253	292.7	279.8	226.2

Sex	M	250	43 (17.2)	146,5	146,4	186.3	106.7
		R: 120	R: 24	72	67.9	90.5	53.5
		U: 13	U: 19	74.5	78.5	95.8	53.2
	F	210	46 (21.9)	128	116.0	159.5	96.5
		R: 95	R: 25	60	49.5	73.5	46.5
		U: 115	U: 21	68	66.5	86.1	49.9
	T	460	89 (19.3)	274.5	262.3	345.8	203.2

Resident	R	215	49 (22.8)	132	117.4	163.9	100.1
		M: 120	M: 24	72	67.9	90.5	53.5
		F: 95	F: 25	60	49.5	73.5	46.5
	U	245	40 (16.3)	142.5	145	181.9	103.1
		M: 130	M: 19	74.5	78.5	95.8	53.2
		F: 115	F: 21	68	66.5	86.1	49.9
	T	460	89 (19.3)	274.5	262.3	345.8	203.2

T : total number; X̄ = sample means (average); SD: standard deviation; CI : confidence interval.

## Data Availability

Data are available on request.
